# Effects of Common Pesticides on Prostaglandin D2 (PGD2) Inhibition in SC5 Mouse Sertoli Cells, Evidence of Binding at the COX-2 Active Site, and Implications for Endocrine Disruption

**DOI:** 10.1289/ehp.1409544

**Published:** 2015-09-11

**Authors:** Subramaniam Kugathas, Karine Audouze, Sibylle Ermler, Frances Orton, Erika Rosivatz, Martin Scholze, Andreas Kortenkamp

**Affiliations:** 1 Institute of Environment, Health and Societies, Brunel University London, Uxbridge, United Kingdom; 2 Molécules Thérapeutiques in silico, Université Paris Diderot-Inserm UMR-S973, Paris, France; 3 Biosciences, College of Life and Environmental Sciences, University of Exeter, Exeter, United Kingdom; 4 Institute of Chemical Biology, Imperial College London, London, United Kingdom

## Abstract

**Background:**

There are concerns that diminished prostaglandin action in fetal life could increase the risk of congenital malformations. Many endocrine-disrupting chemicals have been found to suppress prostaglandin synthesis, but to our knowledge, pesticides have never been tested for these effects.

**Objectives:**

We assessed the ability of pesticides that are commonly used in the European Union to suppress prostaglandin D2 (PGD2) synthesis.

**Methods:**

Changes in PGD2 secretion in juvenile mouse Sertoli cells (SC5 cells) were measured using an ELISA. Coincubation with arachidonic acid (AA) was conducted to determine the site of action in the PGD2 synthetic pathway. Molecular modeling studies were performed to assess whether pesticides identified as PGD2-active could serve as ligands of the cyclooxygenase-2 (COX-2) binding pocket.

**Results:**

The pesticides boscalid, chlorpropham, cypermethrin, cyprodinil, fenhexamid, fludioxonil, imazalil (enilconazole), imidacloprid, iprodione, linuron, methiocarb, o-phenylphenol, pirimiphos-methyl, pyrimethanil, and tebuconazole suppressed PGD2 production. Strikingly, some of these substances—o-phenylphenol, cypermethrin, cyprodinil, linuron, and imazalil (enilconazole)—showed potencies (IC50) in the range between 175 and 1,500 nM, similar to those of analgesics intended to block COX enzymes. Supplementation with AA failed to reverse this effect, suggesting that the sites of action of these pesticides are COX enzymes. The molecular modeling studies revealed that the COX-2 binding pocket can accommodate most of the pesticides shown to suppress PGD2 synthesis. Some of these pesticides are also capable of antagonizing the androgen receptor.

**Conclusions:**

Chemicals with structural features more varied than previously thought can suppress PGD2 synthesis. Our findings signal a need for in vivo studies to establish the extent of endocrine-disrupting effects that might arise from simultaneous interference with PGD2 signaling and androgen action.

**Citation:**

Kugathas S, Audouze K, Ermler S, Orton F, Rosivatz E, Scholze M, Kortenkamp A. 2016. Effects of common pesticides on prostaglandin D2 (PGD2) inhibition in SC5 mouse Sertoli cells, evidence of binding at the COX-2 active site, and implications for endocrine disruption. Environ Health Perspect 124:452–459; http://dx.doi.org/10.1289/ehp.1409544

## Introduction

Although the importance of androgens as drivers of male sexual differentiation in fetal life is widely recognized (Sharpe 2006), the involvement of prostaglandins in these processes has received comparatively little attention. In the 1980s, Gupta and colleagues presented evidence that prostaglandins play a role in the folding and fusion of the penis and scrotum during sexual development in mice (Gupta and Goldman 1986; Gupta and Bentlejewski 1992). These authors observed that arachidonic acid (AA), a precursor of prostaglandins, can reverse the demasculinizing effects of the estrogen receptor agonist estradiol and the androgen receptor antagonist cyproterone during days 11–14 of gestation, the period during which sex differentiation takes place in mice. This reversal could be prevented by coadministration of the analgesics indomethacin and aspirin, both of which inhibit the cyclooxygenase (COX) reaction that produces prostaglandins. Gupta and Bentlejewski (1992) concluded that testosterone drives embryonal sex differentiation by inducing the action of key enzymes of the AA cascade: specifically, phospholipases and COX isoforms. The ability of testosterone to induce enzymes of the arachidonic cascade, including COX, in adult rats was reported by Saito et al. (1986).

In the intervening years, Wilhelm et al. (2007) and Moniot et al. (2009) elucidated the role of prostaglandins as a back-up mechanism for supporting the expression of sex-determining region on chromosome Y (SRY) box containing gene 9 (the *Sox9* gene). As first suggested by Adams and McLaren (2002), prostaglandin D2 is involved in generating a feedback loop to ensure male differentiation of the surrounding gonadal somatic cells. The proposed mechanism for this feedback loop, as detailed by Adams and McLaren (2002), Wilhelm et al. (2007), and Moniot et al. (2009) is as follows: Between gestational days 10.5 and 12, the genital ridge of male mouse embryos produces a wave of Sry, thereby initiating the male differentiation pathway. Sry is a transcription factor that promotes expression of the *Sox9* gene, which drives the differentiation of Sertoli cells in the genital ridge of the mouse. Only fully differentiated Sertoli cells can coordinate the differentiation of all other testicular cell types, including the androgen-producing Leydig cells. Sry and Sox9 upregulate prostaglandin D2 synthase, thereby promoting prostaglandin D2 (PGD2) synthesis and secretion. In turn, PGD2 can act via its prostaglandin D receptor (DP) to upregulate *Sox9* expression in an autocrine and paracrine manner (Adams and McLaren 2002; Moniot et al. 2009; Wilhelm et al. 2007). This PGD2 backup mechanism ensures that cells that have failed to reach a critical threshold of *Sry* expression can still be induced to upregulate *Sox9* and subsequently differentiate into Sertoli cells (reviewed by Koopman 2010). Factors suppressing PGD2 synthesis can therefore be expected to disrupt this backup mechanism, although direct empirical evidence for this idea is lacking. The importance of prostaglandin signaling for normal testis descent recently came to light with the demonstration that mice in which PGD2 synthase was knocked out exhibited unilateral cryptorchidism (Philibert et al. 2013).

The possibility that endocrine disruption might occur through suppression of prostaglandin signaling was suggested as early as 1997, when 1,1-*bis*-(4-chlorophenyl)-2,2-dichloroethene (DDE)–induced eggshell thinning in raptors was linked to direct inhibition of prostaglandin synthesis in the shell gland mucosa, thereby disturbing calcium metabolism (Lundholm 1997). However, few studies had examined prostaglandin signaling as a target for endocrine disruption in mammalian organisms until Kristensen et al. (2011a, 2011b) observed a structural similarity between the COX inhibitor aspirin and certain phthalates, which are well-known endocrine disruptors. These authors demonstrated that phenolic compounds (phthalates, benzophenones, parabens, and alkyl phenols) and a variety of analgesics (paracetamol/acetaminophen, aspirin, ibuprofen, indomethacin) could suppress PGD2 and prostaglandin E2 (PGE2) synthesis in a mouse Sertoli cell line model (SC5 cells), in human mast cells, and in *ex vivo* isolated rat testes. Inhibition of COX enzymes was identified as the likely mode of action for these effects.

The possible human health consequences of suppressing prostaglandins during male sexual differentiation came to light in five epidemiological studies showing that the use of analgesics such as paracetamol by pregnant women towards the end of the first trimester and early in the second trimester (the proposed window of sexual differentiation in humans) is associated with an increased risk of testis maldescent (cryptorchidism) in their sons (Berkowitz and Lapinski 1996; Jensen et al. 2010; Kristensen et al. 2011a; Philippat et al. 2012; Snijder et al. 2012). COX inhibitors such as paracetamol and aspirin were shown to affect the androgen-dependent anogenital index (a biomarker of insufficient androgen action) in the male offspring of rats dosed with paracetamol throughout gestation (Kristensen et al. 2011a).

Evidence from prior research suggests that suppression of prostaglandin synthesis in fetal life might have adverse effects on human health outcomes. We therefore investigated whether chemicals showing structural motifs more varied than those of the phenolics studied by Kristensen et al. (2011b) are also capable of inhibiting PGD2 synthesis. We recently reported that a number of widely used pesticides have androgen receptor antagonist potential *in vitro* and that these chemicals can act in concert when present as mixtures as well as in combination with other antiandrogenic chemicals (Orton et al. 2011, 2012, 2014). Because mouse SC5 cells secrete higher levels of PGD2 than of PGE2 (Kristensen et al. 2011b), we tested these pesticides for their ability to suppress PGD2 and investigated whether a propensity to block the androgen receptor might be related to PGD2-suppressing properties. We also conducted studies to determine the likely mode of action of pesticides identified as active in the SC5 assay, and we performed molecular modeling studies of binding to the COX-2 active site.

## Materials and Methods

*Selection of pesticides.* We selected 19 pesticides (azoxystrobin, boscalid, chlorpropham, chlorpyrifos, cypermethrin, cyprodinil, fenhexamid, fludioxonil, glyphosate, imazalil (enilconazole), imidacloprid, malathion, mancozeb, *o*-phenylphenol (OPP), pirimiphos-methyl, prochloraz, propamocarb, pyrimethanil, and thiabendazole) for in-depth study. Owing to evidence of their antiandrogenicity (Orton et al. 2011; Blystone et al. 2007), we added dimethomorph, iprodione, linuron, methiocarb, and tebuconazole; thus, we selected to investigate a total of 24 pesticides for their PGD2-suppressing properties using an SC5 mouse juvenile Sertoli cell assay (SC5 assay).

*Chemicals.* Dimethomorph and methiocarb were purchased from Greyhound Chromatography and Allied Chemicals (all > 98.7% pure); and all other pesticides (all > 97% pure) were purchased from Sigma Aldrich. Ethanol (> 99.7% purity) was obtained from VWR International Ltd. All test compounds were dissolved in ethanol to prepare stock solutions (20 mM) for use in the SC5 assay. Solubilization of mancozeb and imidacloprid was achieved via sonication.

*SC5 assay for PGD2 inhibition.* SC5 mouse juvenile Sertoli cells (Hofmann et al. 1992) were kindly provided by E. Raipert-de Meyts of the Copenhagen Rigshospitalet, Denmark. We used the assay protocol described by Kristensen et al. (2011b) with slight modifications. Briefly, SC5 cells were cultured in Dulbecco’s modified Eagle’s medium (DMEM; high glucose; Sigma) with 10% fetal calf serum (FCS; Invitrogen Ltd.), 100 U/mL penicillin, 100 μg/mL streptomycin, and 1 mM L-glutamine (all from Sigma) at 37°C and 5% CO_2_. Cells were routinely passaged twice per week, and only phthalate-free polystyrene flasks (Helena BioSciences) and 24-well plates (Corning; VWR) were used. Before each experiment, SC5 cells were seeded in 24-well plates (50,000 cells per well in 500 μL medium). On the following day, all of the medium was removed and 400 μL fresh medium with test compound was added. Each plate contained a solvent control (0.5% ethanol in medium) and a positive control (1% octylphenol ethoxylate, Sigma) that served as a reference for cytotoxicity potentially induced by the test compounds. All pesticides were tested in three independent experiments and at eight different concentrations; ibuprofen was included in each experiment as a positive control for PGD2 suppression. Plates were incubated at 37°C for 24 hr in an atmosphere of 5% CO_2_. At the end of the exposure period, 100 μL medium was removed for PGD2 measurements and kept on ice. PGD2 levels were determined using a prostaglandin D2-MOX enzyme immunoassay (EIA; Cayman Chemicals) according to the manufacturer’s instructions. Plates were read at a wavelength of 405 nm with a reference wavelength of 620 nm (Spectramax 340 PC). PGD2 concentrations in samples from treated cell cultures were determined using the linear range of a standard curve and are presented as percentages of the PGD2 levels measured in the solvent controls. To correct for inter-experiment data variability and to allow for regression analysis of pooled data, the PGD2 readings for the tested compounds on each EIA plate were expressed as percentages of the average of the values obtained from the solvent controls.

*Cytotoxicity determination.* After aliquots of supernatant medium were removed for PGD2 determination, the plated SC5 cells were subjected to cytotoxicity testing with a modified version of the 3-(4,5-dimethylthiazol-2-yl)-2,5-diphenyltetrazolium bromide (MTT) assay (Mosmann 1983), as described by Orton et al. (2012). Data were normalized to both the solvent controls and the positive controls. Readings of normalized absorbance values < 80% of solvent controls were considered cytotoxic.

*Studies of the mode of PGD2 inhibitory action.* Time series studies were conducted in which SC5 cells were exposed to ibuprofen (130 nM), aspirin (3,400 nM), and OPP (175 nM) for 5 min, 15 min, 30 min, 1 hr, 2 hr, 3 hr, 6 hr, and 24 hr (three experiments performed in duplicate). The selected concentrations corresponded to half-maximal suppression of PGD2 levels (IC50) as determined in concentration–response analyses for the full duration of exposure (24 hr) in the SC5 assay.

We assessed the mode of action of OPP by comparing its effects with those of ibuprofen, aspirin, and the phospholipase A_2_ (PLA_2_) inhibitor MJ33 in terms of the reversibility of PGD2 inhibition and the influence of arachidonic acid (AA) on PGD2 levels. SC5 cells were exposed to ibuprofen (10 μM), aspirin (100 μM), OPP (10 μM), or MJ33 (100 μM, Sigma) for 4 hr. After 4 hr of exposure, the medium was processed for PGD2 measurements as described previously. Some cultures received one of the following treatments for a further 2 hr: *a*) the treatment medium was removed and the cells were washed with Hanks’ Balanced Salt Solution (HBSS), after which 400 μL fresh culture medium (without any of the treatment chemicals) was added; *b*) the same as in *a*), but the added culture medium (400 μL) contained 10 μM AA; or *c*) a small volume of AA solution was added to the treatment medium containing the test chemicals to a final concentration of 10 μM AA. At the end of the experiments, PGD2 concentrations were measured as described previously.

The influence of AA supplementation on PGD2 levels was also studied using a range of pesticides. After exposing SC5 cells to increasing concentrations of AA (between 0.3 and 100 μM) for 24 hr, we chose to use 10 μM AA for experiments with test chemicals. We exposed SC5 cells to varying concentrations of ibuprofen or OPP for 22 hr without AA and then added AA (10 μM) for another 2 hr before terminating the experiment. PGD2 levels were determined as described above. The same protocol was used for nine pesticides [boscalid, chlorpropham, cypermethrin, cyprodinil, imazalil (enilconazole), imidacloprid, linuron, pirimiphos-methyl, and tebuconazole], where each pesticide was tested at levels producing 80% (IC80) PGD2 suppression relative to the solvent controls. These (highly effective) concentrations were chosen to provide unambiguous evidence of the effects of AA supplementation.

*Molecular modeling studies of binding to the COX-2 active site.* We conducted computer modeling studies using Molecular Operating Environment (MOE) software (v2011.10; Chemical Computing Group Inc., Montreal, Canada). The crystal structure of murine COX-2, which is very similar to human COX-2 (Kurumbail et al. 1996), was obtained from the Research Collaboratory for Structural Bioinformatics (RCSB) Protein Data Bank at 2.9 Å resolution [accession no. 1PXX; primary data provided by Rowlinson et al. (2003)]. The monomer structure (chain A) was used for molecular docking, with water molecules kept within the model. Docking studies were performed by placing selected pesticides within the active site of COX-2 in a position allowing the best match with hydrogen bonding to Ser530 and Tyr385. A maximum of 30 positions (poses) were assessed for each chemical, which proved sufficient for making judgements about binding specificity. We used the Andrews p*K*i score as an estimate of how well the selected pose(s) of the test chemical fit to an average binding site. The quality of the fit was computed from the test pesticide’s structure alone and was based on the physicochemical properties (e.g., number of hydrogen bond donors/acceptors, distances) without considering features of the COX-2 binding pocket. We also calculated a predicted p*K*i score based on computed positions that the ligand can assume in the COX-2 active site. The predicted p*K*i score takes into account the structural conformation of the test chemical and the properties of the binding site of COX-2. The best poses were selected based on energy minima and interactions within the binding site. By comparing the Andrews p*K*i score and the predicted p*K*i, we judged whether the pesticide used all of its potential energy for binding in a particular position. If the predicted p*K*i was substantially greater than the Andrews p*K*i score, the ligand was specific to COX-2.

*Statistical analysis.* For all concentration–response analyses, experiments were repeated three times; the data from the same test compound were pooled, and nonlinear regression analyses were performed using the best-fit approach (Scholze et al. 2001). We used a variety of nonlinear regression models that were fitted independently to the same data set. The best-fitting model was selected using a statistical goodness-of-fit criterion. All statistical analyses were performed using SAS statistical software (SAS Institute Inc., Cary, NC, USA). From the regression analyses, we derived estimates of IC50 (the concentration of test agent that inhibits the PGD2 levels by 50% compared with the solvent controls).

## Results

**Figure 1:**
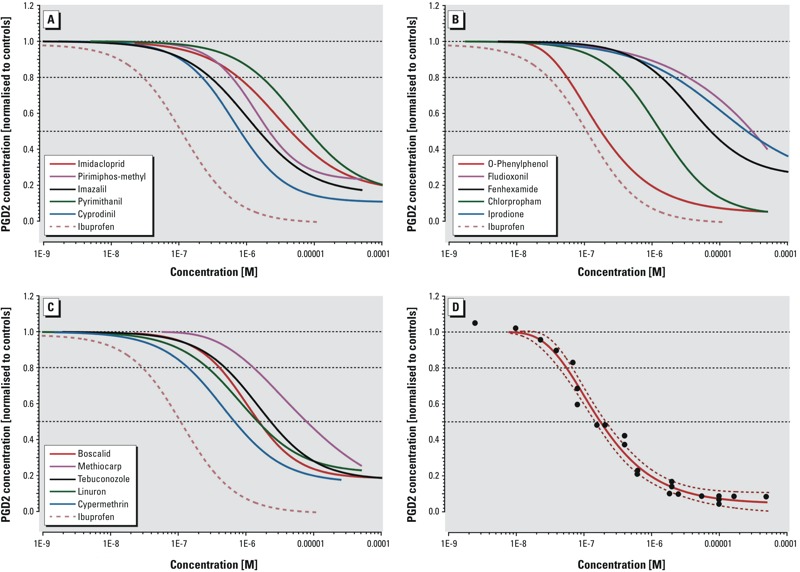
Suppression of prostaglandin D2 synthesis by pesticides in the mouse Sertoli cell assay. (*A*–*C*) Best-fitting regression models for suppression of prostaglandin D2 (PGD2) synthesis in SC5 (mouse Sertoli) cells after 24-hr exposure to selected pesticides (3 replicates). Pesticides are grouped according to their exposure in the European Union (Orton et al. 2011) in *A*, *B*, and *C* as high, medium, and low exposure, respectively. All data were normalized to the responses of solvent controls. Ibuprofen was chosen as the positive control (dashed line in *A*–*C*). (*D*)**Responses (black dots) and best-fitting regression model with 95% confidence belts (dashed and solid red lines) for *o*-phenylphenol.

*Suppression of prostaglandin synthesis in the SC5 assay.* We selected 24 pesticides (for chemical structures, see Supplemental Material, Figure S1) to be tested in the present study. After converting the PGD2 readings for the tested compounds on each EIA plate to percentages of the average values for solvents, 15 of the initial 24 [boscalid, chlorpropham, cypermethrin, cyprodinil, fenhexamid, fludioxonil, imazalil (enilconazole), imidacloprid, iprodione, linuron, methiocarb, OPP, pirimiphos-methyl, pyrimethanil, and tebuconazole] were found to inhibit PGD2 synthesis in a concentration-dependent manner in the SC5 assay, as shown in Figure 1A–C (data and regression models from three independent experiments for groups of pesticides and ibuprofen) and in Figure 1D (for OPP); see also Supplemental Material, Figure S2 (for the remaining compounds). The concentration–response relationship for the known nonsteroidal anti-inflammatory drug ibuprofen is also shown.

With an IC50 of 175 nM for PGD2, the fungicide OPP was the most potent of the selected pesticides and was nearly as potent as the analgesic ibuprofen (IC50: 128 nM), which we used as a positive control (Table 1 and Figure 1). Cypermethrin, an insecticide, and cyprodinil, another fungicide, were the next most potent agents, with IC50 values of 678 nM and 803 nM, respectively, followed by the herbicide linuron (1,490 nM) and the fungicide imazalil (enilconazole) (1,510 nM). The IC50 values of the other tested pesticides were between 1,340 nM and 30.2 μM (Table 1; see also Supplemental Material, Figure S2). The concentrations of test agents used in the SC5 assay were used in the MTT assay to assess the cytotoxicity of the pesticides. Cytotoxicity in the concentration ranges shown to be associated with suppression of PGD2 synthesis was not observed (see Supplemental Material, Figure S3). Azoxystrobin, chlorpyrifos, dimethomorph, glyphosate, malathion, mancozeb, prochloraz, propamocarb, and thiabendazole, listed as “inactive” in Table 1, did not inhibit PGD2 synthesis at any of the tested concentrations, which ranged between 0.1 nM and 0.1 mM.

**Table 1:** List of chemicals tested for PGD2 synthesis inhibition in mouse Sertoli cells.

Pesticide	Use	PGD2 inhibition IC50 (nM)	AR antagonism IC50 (nM); Orton et al. 2011
Mancozeb	Fungicide	Inactive	Inactive
Cyprodinil	Fungicide	803	28,100
Imazalil (enilconazole)	Fungicide	1,510	8,300
Pirimiphos-methyl	Insecticide	2,140	17,100
Pyrimethanil	Fungicide	8,270	98,600
Thiabendazole	Fungicide	Inactive	Inactive
Malathion	Insecticide	Inactive	Inactive
Imidacloprid	Insecticide	4,450	Inactive
Fludioxonil	Fungicide	30,200	2,620
Azoxystrobin	Fungicide	Inactive	Inactive
Fenhexamid	Fungicide	7,370	7,080
Iprodione	Fungicide	25,200	Inactive
Propamocarb	Fungicide	Inactive	Inactive
Chlorpyrifos	Insecticide	Inactive	Inactive
Chlorpropham	Herbicide	1,340	19,200
*o*-Phenylphenol	Fungicide	175	9,570
Cypermethrin	Insecticide	678	Inactive
Boscalid	Fungicide	1,550	Not tested
Glyphosate	Herbicide	Inactive	Inactive
Prochloraz	Fungicide	Inactive	6,030
Methiocarb	Insecticide	7,850	14,800
Tebuconazole	Fungicide	2,320	8,060
Dimethomorph	Fungicide	Inactive	940
Linuron	Herbicide	1,490	6,630
Ibuprofen	NSAID	128	Inactive
Aspirin	NSAID	3,426	Inactive
Abbreviations: AR, androgen receptor; IC50, concentration at half-maximal suppression; NSAID, nonsteroidal antiinflammatory drug; PGD2, prostaglandin D2. IC50 values are shown for pesticides found to inhibit PGD2 synthesis.			

*Studies of the mode of PGD2-inhibitory action.* To obtain more information about the mode of action by which pesticides inhibited PGD2 synthesis, we initially conducted detailed experiments only with OPP, the most potent of the pesticides studied herein. We compared its effects with those of ibuprofen, aspirin, and the PLA_2_ inhibitor MJ33. Aspirin suppresses PGD2 synthesis by irreversibly inactivating COX-1 and COX-2 through relatively rapid, covalent modification of the COX active site (acetylation of Ser530), thereby blocking AA binding. Ibuprofen inhibits PGD2 synthesis through competitive enzyme inhibition by reversibly binding to the AA binding site of both COX isoforms (reviewed by Blobaum and Marnett 2007). To investigate whether OPP suppressed PGD2 synthesis reversibly in a manner similar to that of ibuprofen, or in an irreversible fashion akin to that of aspirin, we began by conducting time–course studies using concentrations that have been shown to induce half-maximal PGD2 suppression after 24 hr (175 nM OPP, 128 nM ibuprofen, and 3,400 nM aspirin). After 60 min of incubation, aspirin reduced PGD2 levels to approximately 50% of control levels. OPP and ibuprofen required at least 120 min to achieve the same effect and showed almost identical time courses. By 4 hr, PGD2 levels had plateaued in all cases (see Supplemental Material, Figure S4).

**Figure 2:**
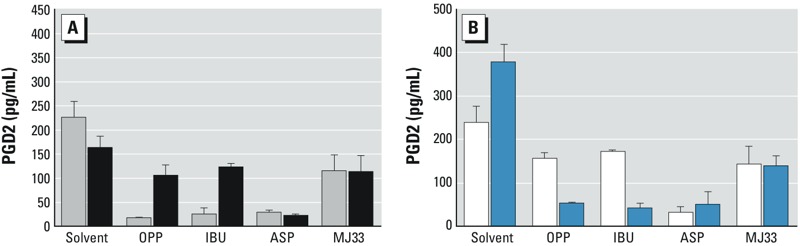
The mode of action of OPP in suppressing prostaglandin D2 synthesis. Abbreviations: ASP, aspirin; IBU, ibuprofen; MJ33, phospholipase A_2_ inhibitor; OPP, *o*-phenylphenol. (*A*) Prostaglandin D2 (PGD2) levels (pg/mL) after exposure of SC5 cells to solvent, OPP (10 μM), IBU (10 μM), ASP (100 μM), and MJ33 (100 μM) for 4 hr (gray bars), or for 4 hr, followed by medium exchange, a wash step, and further incubation for 2 hr with fresh medium without test compounds (black bars). (*B*) PGD2 levels in SC5 cells after exposure to the same agents as in (*A*) for 4 hr, followed by medium exchange, a wash step, and addition of arachidonic acid (AA; 10 μM) without test chemicals for a further 2 hr (white bars). Blue bars show PGD2 levels after treatment for 4 hr followed by addition of AA to the medium to give a final concentration of 10 μM and treatment with test chemicals for a further 2 hr. Bars show the means of three independent experiments performed in duplicate; error bars represent the standard deviations. All differences were statistically significant (*t*-test, *p *< 0.01) except those shown for ASP and MJ33.

We sought to further corroborate the mode of action of OPP by comparing it with the PLA_2_ inhibitor MJ33. We treated SC5 cells for 4 hr with OPP (10 μM), ibuprofen (10 μM), aspirin (100 μM), or MJ33 (100 μM), removed the medium, briefly washed the cells, and incubated them with fresh medium (not containing OPP, ibuprofen, aspirin, or MJ33) for another 2 hr. For OPP and ibuprofen, the replenishment with fresh medium led to increases in PGD2 levels relative to cultures that had not received media changes at the end of the 4-hr exposure period (Figure 2A, black bars), suggesting that the inhibition of COX enzymes was no longer effective. For MJ33, similar PGD2 levels were reached after media replenishment. In contrast, cells treated with aspirin remained unable to produce PGD2 because of the irreversibility of aspirin’s action (Figure 2A).

Next, we conducted experiments to assess whether the observed suppression of PGD2 synthesis resulted from blocking the release of arachidonic acid (AA) from phospholipids through inhibition of PLA_2_ (as with the PLA_2_ inhibitor MJ33) or by inhibition of COX isoforms (as with ibuprofen and aspirin). To distinguish between these possibilities, we studied the effects of AA supplementation on PGD2 levels. In cells exposed to OPP, ibuprofen, or MJ33, removal of the treatment medium followed by washing and adding fresh medium with AA (10 μM) but no test chemical gave rise to PGD2 levels of a magnitude similar to those observed before exposure, indicating that AA could once again be used for PGD2 production after changes of medium (Figure 2B, white bars). However, this was not the case for aspirin, where PGD2 levels remained low relative to those for the solvent controls, indicating that utilization of AA continued to be blocked despite the addition of AA after removal of the drug (Figure 2B, white bars). Finally, we added AA (10 μM) to cultures that had been treated with all agents for 4 hr and incubated them for a further 2 hr, but this time in the presence of treatment agents. Cells exposed to OPP, ibuprofen, and aspirin (Figure 2B, blue bars) produced much smaller additional amounts of PGD2 than cells that underwent the 4-hr treatment with wash-out and AA replenishment without test chemicals (Figure 2B, white bars). The ability of cells exposed to OPP, ibuprofen, or aspirin to utilize AA for PGD2 synthesis was compromised, presumably because of COX inhibition. In contrast, cells exposed to the PLA_2_ inhibitor MJ33 were able to continue with PGD2 synthesis. The inability of AA to overcome the PGD2-inhibitory effects in the presence of OPP indicates that the pesticide acts by inhibiting COX enzymes in a fashion similar to ibuprofen. Alternatively, OPP could disrupt the downstream reactions that isomerize prostaglandin H2 to PGD2 and other prostaglandins. Had the inhibition target of OPP been upstream of the COX reaction and involved the release of AA from phospholipids by PLA_2_, the addition of AA would have led to a recovery of PGD2 levels, as was observed for the PLA_2_ inhibitor MJ33.

Next, we tailored our AA supplementation studies to the treatment regimen used to record the concentration–response relationships with pesticides over a 24-hr exposure period. Accordingly, we treated SC5 cells with test chemicals for 22 hr and then added AA for an additional 2 hr. Under these conditions, AA was expected to stimulate PGD2 levels only if COX enzymes were active, either because the test chemicals act upstream of COX (e.g., on PLA_2_) because they have degraded and thus lost their ability to inhibit COX isoforms, or because COX expression increased during the long incubation period. To establish an optimal AA concentration for these experiments, we studied the influence of increasing concentrations of AA on PGD2 levels. After 24 hr, 10 μM AA produced approximately 300% increases in PGD2 levels relative to solvent controls (see Supplemental Material, Figure S5). This concentration of AA was selected for coincubation with PGD2-active pesticides. When cells were incubated with ibuprofen and OPP for 22 hr followed by AA (10 μM) supplementation for another 2 hr, no changes in PGD2 levels were observed (see Supplemental Material, Figure S5), again suggesting that OPP suppresses PGD2 synthesis by inhibiting COX.

**Figure 3:**
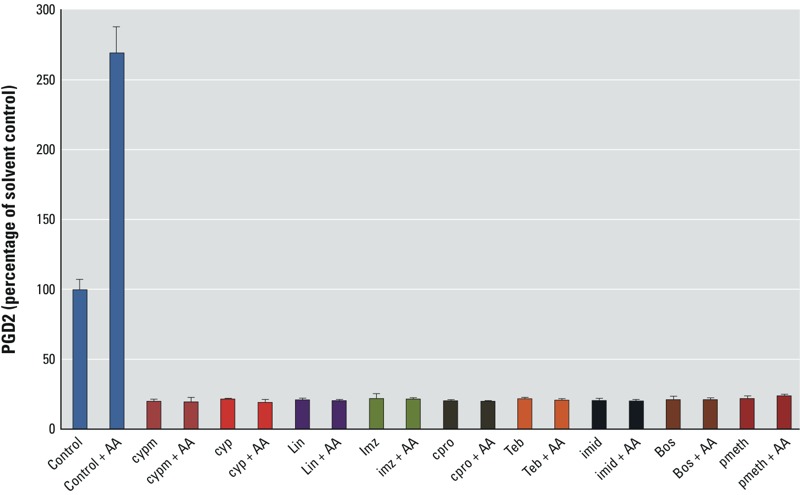
The influence of AA supplementation on the PGD2-suppressing effects of selected pesticides. Abbreviations with IC80 given in parentheses: AA, arachidonic acid; bos, boscalid, (20 μM); cpro, chlorpropham, (10 μM); cyp, cyprodinil, (10 μM); cypm, cypermethrin, (10 μM); imid, imidacloprid, (50 μM); imz, imazalil (enilconazole), (10 μM); lin, linuron, (10 μM); pmeth, pirimiphos-methyl, (50 μM); teb, tebuconazole, (50 μM). PGD2 levels in mouse Sertoli cells exposed for 24 hr to pesticides at concentrations producing 80% suppression of PGD2 synthesis, with and without arachidonic acid (AA) supplementation. Comparisons are made with cells that received AA (10 μM) for 2 hr after 22 hr of exposure to pesticide. The means of 3 experiments are shown; error bars represent standard deviations.

According to our concentration–response analyses (Figure 1 and Table 1; see also Supplemental Material, Figure S2), the next nine most potent PGD2-active agents after OPP were boscalid, chlorpropham, cypermethrin, cyprodinil, imazalil (enilconazole), imidacloprid, linuron, pirimiphos-methyl, and tebuconazole. These agents were chosen to analyze the effects of AA supplementation (for 2 hr) following 22 hr of exposure. Each of these pesticides was administered to SC5 cells at a concentration producing 80% PGD2 suppression relative to solvent controls. In all cases, the addition of AA had no effects on PGD2 suppression (Figure 3), again suggesting that the mode of action is inhibition of COX isoforms. We attempted to corroborate this directly by performing COX activity assays (Cayman 700200, 760151) but found that these assays were insufficiently sensitive for the number of cells used in the SC5 assay. We therefore conducted molecular modeling studies of binding to the COX-2 active site to further investigate the likelihood of COX inhibition.

*Molecular modeling studies of binding to the COX-2 active site.* The binding site of COX enzymes is a hydrophobic channel that has hydrogen bonding sites at its mouth (Tyr355 and Arg120) as well as at its base (Tyr385); another possible target for hydrogen bonding is the acetyl salicylic acid acetylation site (Ser530) positioned below Tyr385 at the base of the channel (Kurumbail et al. 1996; Luong et al. 1996; Picot et al. 1994). We performed molecular modeling to determine whether pesticides capable of suppressing PGD2 would fit into the COX-2 binding site. We approached this investigation by comparing the Andrews mean p*K*i scores for a given compound, which estimates docking in a random binding site, with predicted p*K*i scores obtained by positioning a pose within the COX-2 binding site in a way that allowed the best match with its hydrogen-bonding sites. If the mean predicted dissociation constants (predicted p*K*i scores) of the binding of the PGD2-active pesticides were higher than the Andrews mean p*K*i scores, as with ibuprofen, these compounds could be fitted in the ligand-binding pocket of COX-2. As shown in Table 2, this was the case for 13 of the 15 PGD2-active pesticides. The exceptions were iprodione and imidacloprid, where the Andrews mean p*K*i scores were higher than the predicted p*K*i scores.

**Table 2:** Results of modeling studies of docking of pesticides into the active site of COX-2.

Chemical	Andrews mean p*K*i^*a*^	Predicted p*K*i^*b*^	Hydrogen bonds	Hydrophobic interaction	Difference between predicted p*K*i and Andrews mean p*K*i
Ibuprofen	–5.26	4.35	0.56	3.06	9.61
*o*-Phenylphenol	–3.29	4.94	0.13	3.97	8.23
Cypermethrin	0.43	9.14	0.2	8.83	8.71
Cyprodinil	–1.68	4.53	0	4.13	6.21
Linuron	–0.87	4.92	0.64	3.44	5.79
Imazalil (enilconazole)	–1.46	4.17	0.24	3.82	5.63
Chlorpropham	–4.61	4.12	0.19	3.09	8.73
Boscalid	3.07	6.45	0.23	6.25	3.38
Tebuconazole	0.36	4.72	0	4.61	4.36
Pirimiphos-methyl	–5.05	2.63	1.01	1.51	7.68
Imidacloprid	5.42	2.62	0.15	1.77	–2.80
Methiocarb	–4.17	3.71	0.49	2.67	7.88
Fenhexamid	2.49	6.41	0.42	5.44	3.92
Pyrimethanil	–2.34	4.24	0	3.26	6.58
Iprodione	6.15	4.62	0.52	3.70	–1.53
Fludioxonil	0.21	4.15	0.01	3.44	3.94
Abbreviations: COX-2, cyclooxygenase 2; p*K*i, binding affinity. ^***a***^Andrews p*K*i is an estimate of how well the selected pose of the docked ligand can bind to the active site of the COX-2 receptor. This value is computed from ligand structure alone, without a receptor. ^***b***^Predicted p*K*i score is the predicted binding of the ligand into the COX-2 active site. The value might vary according to the pose. The pose was selected based on low energy value and interactions within the binding site.					

**Figure 4:**
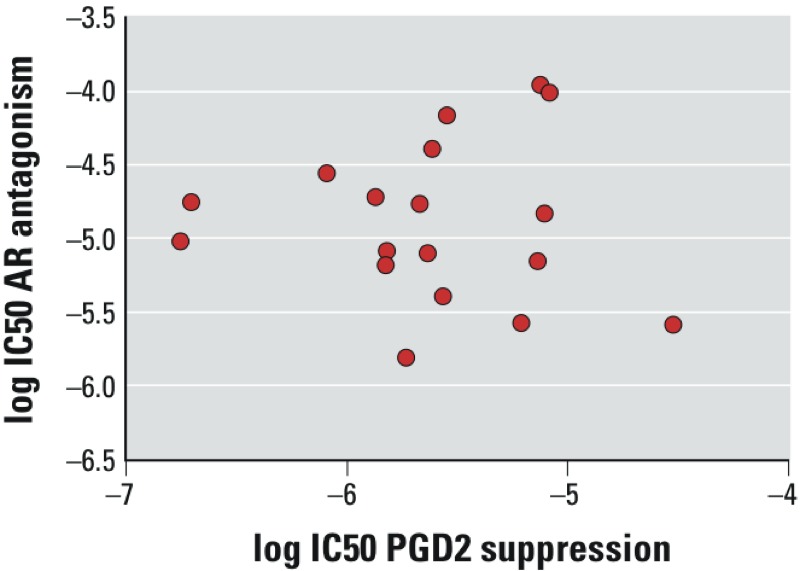
Lack of correlation between the ability to suppress PGD2 synthesis and androgen receptor antagonist potency. Plot showing log IC50 for androgen receptor antagonist potency (*in vitro*) versus log IC50 for PGD2 suppression for ethylparaben, n-propylparaben, n-butylparaben, benzophenone 3, bisphenol A, flutamide, 1,1-bis-(4-chlorophenyl)-2,2-dichloroethene (DDE), cyprodinil, imazalil (enilconazole), pirimiphos-methyl, pyrimethanil, fludioxonil, fenhexamid, chlorpropham, o-phenylphenol, methiocarb, tebuconazole, and linuron. For IC50 values, see Supplemental Material, Table S1. Data are from Ermler et al. (2011), Kristensen et al. (2011b), Orton et al. (2011), and the present study.

*Lack of correlation between the ability to suppress PGD2 and antagonism at the androgen receptor.* Of the 15 pesticides identified as capable of suppressing PGD2 synthesis, 11 were also found to antagonize the androgen receptor *in vitro* (Table 1, and Orton et al. 2011). Only 3 pesticides (cypermethrin, imidacloprid, and iprodione) showed activity in the SC5 assay without also antagonizing the androgen receptor; boscalid was not tested for androgen receptor antagonism. This high concordance prompted us to investigate whether high potency in PGD2 suppression is associated with strong androgen receptor–antagonist potency. By collating data reported elsewhere (Ermler et al. 2011; Kristensen et al. 2011b; Orton et al. 2011), we identified additional chemicals that were capable of both suppressing PGD2 in the SC5 assay and antagonizing the androgen receptor in the MDA-kb2-luc assay (see Supplemental Material, Table S1). However, plots of log IC50 (androgen receptor antagonism) versus log IC50 (PGD2 suppression) did not reveal a correlation between these values, suggesting that the two mechanisms are entirely different and are not related to each other (Figure 4).

## Discussion

We have shown that several pesticides currently authorized for use in the European Union can suppress PGD2 synthesis *in vitro*. Strikingly, some of these substances [OPP, cypermethrin, cyprodinil, linuron, tebuconazole, and imazalil (enilconazole)] showed potencies (IC50s) ranging between 175 and 2,300 nM (Figure 1, Table 1); these values are comparable to those measured for some analgesics intended to inhibit COX enzymes, including ibuprofen (128 nM) and aspirin (5,380 nM).

In principle, these PGD2-suppressing effects can arise from inhibition of multiple elements of the AA cascade, including phospholipase A_2_, COX enzymes that produce PGH2, and the downstream reactions that convert PGH2 into PGD2. Our observation that AA supplementation failed to rescue the PGD2-inhibiting effects of some of our test compounds suggests that the mechanism of action can be narrowed down to COX inhibition and/or to downstream events involving PGH2 conversion to PGD2. Inhibition of PGD2 synthesis could also be a consequence of downregulation of COX enzyme expression, but this possibility has been shown not to apply to various endocrine-disrupting chemicals (Kristensen et al. 2011b). Our results provide further evidence that COX downregulation is not the mode of action of the chemicals tested here: PGD2 suppression by OPP occurs within 4 hr (Figure 2); this time frame is too short to bring about changes in protein levels via alterations in gene expression [typically, 12–24 hr is required for such changes; see, e.g., Silva et al. (2010)]. Within 2 hr of removing the chemical, recovery from the PGD2-suppressing effects was observed; again, this change occurred too rapidly for altered mRNA levels to be responsible.

The outcome of our docking studies also supports inhibition of COX isoforms as the most likely mechanism of action. With the exception of iprodione and imidacloprid, all PGD2-suppressing pesticides could be fitted in the ligand-binding pocket of COX-2. This finding suggests that the PGD2-active pesticides act by sterically hindering AA access to the active site of COX-2, and perhaps to that of COX-1, in a manner similar to the way in which classical nonsteroidal antiinflammatory drugs such as ibuprofen inhibit these enzymes. Irreversible inhibition of COX enzymes, typical of the mode of action of aspirin, was shown not to be relevant with OPP.

Our study shows that chemicals with structural features far more varied than those of the phenolic compounds investigated by Kristensen et al. (2011b) can inhibit PGD2 synthesis. Although the substances identified by Kristensen et al. (2011b) possessed a terminal apolar benzene ring similar to that of aspirin or ibuprofen as a common feature, here, we show that pesticides lacking this structural motif (pirimiphos-methyl, methiocarb, fludioxonil, imidacloprid) can also suppress PGD2 synthesis.

The SC5 assay proved to be a valuable screening tool for revealing the prostaglandin-suppressing properties of chemicals. In view of the importance of prostaglandin signaling in sustaining *Sox9* expression and Sertoli cell differentiation (Adams and McLaren 2002; Moniot et al. 2009; Wilhelm et al. 2007), it can be expected that prostaglandin suppression early in fetal life will contribute to disruption of male sexual differentiation. The strongest evidence for this idea comes from observations of unilateral cryptorchidism in a mouse knockout model for PGD2 synthase (Philibert et al. 2013). Furthermore, shortened anogenital distances were found in male rat offspring after *in utero* exposure to the COX inhibitor paracetamol, together with reduced PGD2 levels in rat fetal explants treated with paracetamol or aspirin (Kristensen et al. 2011a).

However, at the present time, it is unclear whether the pesticides investigated herein will induce adverse effects in experimental animals related to disrupted male sexual differentiation via a PGD2-mediated mode of action; this possibility remains to be demonstrated. Such a demonstration will depend on factors that cannot be captured by the SC5 assay, including timing of exposure, transport, systemic circulation, and metabolism. It is possible that rapid metabolism and other toxicokinetic factors ensure that many of the PGD2-active pesticides do not reach effective tissue levels, thereby preventing adverse effects from materializing *in vivo* at dosages tolerated by the experimental animals. It is also unclear whether the possible adverse developmental effects of prostaglandin suppression that arise from exposure in fetal life are limited to the reproductive system. Several other cell types, including endocrine-active cells in the pancreas and endocrine-active mesenchymal cells such as adipocytes, are derived from the same mesenchymal stem cells as Leydig cells. If these cell types are also susceptible to prostaglandin signaling, effects beyond the reproductive system may arise, but this possibility has remained largely unexplored.

Associations between exposure to mild analgesics in pregnancy and congenital malformations such as cryptorchidism and hypospadias have been reported based on observational epidemiological studies (Jensen et al. 2010; Kristensen et al. 2011a; Philippat et al. 2012; Snijder et al. 2012). However, whether these effects can be attributed solely to COX inhibition is unclear because paracetamol and its metabolites were shown to also suppress production of InsL3, the hormone responsible for the first phase of testis descent (Mazaud-Guittot et al. 2013). Recent studies have shown that PGD2 is involved in suppressing cell invasion and metastasis in testis cancer cells (Shyu et al. 2013; Wu et al. 2012).

We have identified chemicals capable of simultaneously suppressing PGD2 synthesis and blocking the androgen receptor, thereby affecting several mechanisms and modalities relevant to male sexual differentiation. Most of the PGD2-inhibiting pesticides (11 out of 15, Table 1; see also Supplemental Material, Table S1) fall into this category. It will be interesting to assess the consequences of such multiple modes of action *in vivo* because no relevant data regarding the disruption of male sexual differentiation by such multiple modalities are available in the literature. Conversely, the pesticides that have PGD2-suppressing properties but lack androgen receptor–antagonistic effects (cypermethrin, imidacloprid, and iprodione) may provide useful tools for disentangling the relative importance of prostaglandin synthesis inhibition in producing adverse male reproductive effects. The lack of correlation between potency in suppressing PGD2 and antagonizing the androgen receptor (Figure 4) suggests that such dual activities are coincidental and are not linked in any mechanistic way.

OPP, the most potent PGD2-suppressing agent tested herein, is an antimicrobial agent used as a fungicide and sanitizer on, for example, pears and citrus fruits. The available data suggest that exposure to this pesticide is widespread, albeit at low levels. OPP was detected in 40 of 60 different canned beers at concentrations in the low parts-per-billion range (Coelhan et al. 2006); in all human urine samples tested in two studies (Bartels et al. 1997; Ye et al. 2005); in 85% of breast milk samples (Ye et al. 2006); and in 30% of amniotic fluid samples (Bradman et al. 2003) in the United States.

## Conclusion

Taken together, our findings support concerns that chronic exposure to multiple endocrine disruptors that interfere with PGD2 signaling and diminish androgen action, along with episodes of high exposures to mild analgesics during pregnancy, may pose previously unrecognized risks to developing male fetuses. We have recently shown that paracetamol and other antiandrogens can act together to produce demasculinizing effects in male rats after *in utero* exposure (Christiansen et al. 2012). The likely effects on humans are difficult to anticipate at present. The results of these studies support the need to evaluate the effects of pesticides on PGD2 synthesis *in vivo*.

## Supplemental Material

(2.4 MB) PDF
