# Supplementary material for: Effects of Common Pesticides on Prostaglandin D2 (PGD2) Inhibition in SC5 Mouse Sertoli Cells, Evidence of Binding at the COX-2 Active Site, and Implications for Endocrine Disruption
Source: Environ Health Perspect. 2015 Sep 11;124(4):452–9. doi: 10.1289/ehp.1409544 (PMC4829986; doi:10.1289/ehp.1409544)
Supplement: (2.4 MB) PDF [file ehp.1409544.s001.acco.pdf]

Environ Health Perspect
DOI: 10.1289/ehp.1409544

Note to Readers: *EHP* strives to ensure that all journal content is accessible to all readers. However, some figures and Supplemental Material published in *EHP* articles may not conform to 508 standards due to the complexity of the information being presented. If you need assistance accessing journal content, please contact ehp508@niehs.nih.gov. Our staff will work with you to assess and meet your accessibility needs within 3 working days.

Supplemental Material

Effects of Common Pesticides on Prostaglandin D2 (PGD2) Inhibition in SC5 Mouse Sertoli Cells, Evidence of Binding at the COX2 Active Site, and Implications for Endocrine Disruption

Subramaniam Kugathas, Karine Audouze, Sibylle Ermler, Frances Orton, Erika Rosivatz, Martin Scholze, and Andreas Kortenkamp

Table of Contents

Figure S1: Chemical structures of tested pesticides

Pesticides inhibiting PGD2 synthesis in SC5 cells

Pesticides tested but not inhibiting PGD2 synthesis in SC5 cells

Common NSAIDs

Figure S2: Suppression of PGD2 synthesis by pesticides in the mouse Sertoli cell assay. The graphs show data, best-fitting models (solid line) and 95% confidence belts (dashes line) for PGD2 synthesis inhibiting responses in SC5 (mouse Sertoli) cells after 24 hours exposure (3 replicates). All data were normalised to those of solvent controls.

Figure S3: Lack of cytotoxicity of test agents in the concentration ranges associated with PGD2 suppression. Shown are MTT responses (absorbance at 570 nm) in SC5 cells normalised to solvent-treated controls (absorbance readings in controls were set to 100%). The range associated with PGD2 suppression is depicted by the grey boxes. A clear downward trend in responses was not seen over the concentration ranges investigated.

Figure S4: Time course of PGD2 suppression in SC5 cells by OPP, ibuprofen and aspirin. SC5 cells were exposed to ibuprofen (IBU), aspirin (ASP) and o-phenylphenol (OPP) at their respective IC50 concentrations (128 nM, 3426 nM and 175 nM of ibuprofen, aspirin and OPP, respectively) for the indicated time periods, and PGD2 concentrations measured. For each time point, PGD2 levels (average of 3 experiments performed in duplicate) are expressed as percentage of solvent controls. The last time point is 24 hours.

Figure S5: Effect of AA supplementation on the inhibition of PGD2 synthesis by ibuprofen and o-phenylphenol. PGD2 synthesis in mouse Sertoli cells after 24 h exposure to ibuprofen (black solid line) and o-phenylphenol (black dashed line) without AA. Supplementation of AA (10 μM) during the last 2 hours of the 24 h exposure duration, with ibuprofen (blue solid line) or o-phenylphenol (blue dashed line). The inset figure (red line) shows the dose response curve of AA stimulation of PGD2 synthesis. The graphs show mean responses (dots, n=3) and their best-fitting regression models. Data were normalised against solvent controls.

Table S1: Different endocrine disrupting compounds and their IC_{50} values in PGD2 inhibition and AR-antagonism assays. Values for ethylparaben, *n*-propylparaben, *n*-butylparaben, benzophenone 3, bisphenol A, flutamide and *p,p'*-DDE (PGD2 inhibition) were obtained from Kristensen et al. (2011 b), and AR antagonism from Ermler et al. (2011). The AR antagonism data for all remaining chemicals are from Orton et al. (2011), those for PGD2 inhibition are data reported here (shown in bold).

References

Figure S1: Chemical structures of tested pesticides

Pesticides inhibiting PGD2 synthesis in SC5 cells

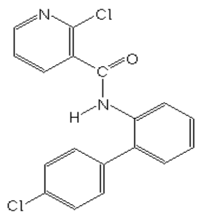

Boscalid

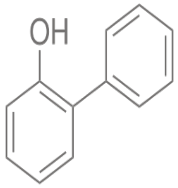

O-phenylphenol

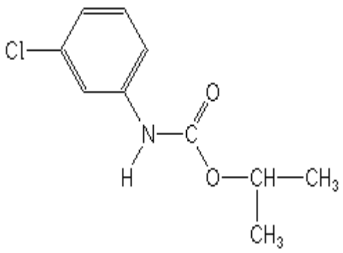

Chlorpropham

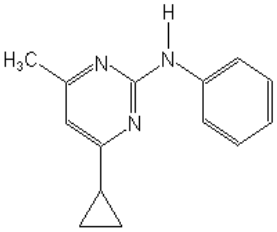

Cyprodinil

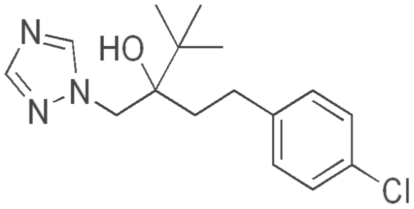

Tebuconazole

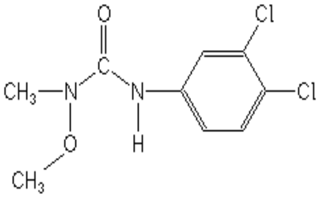

Linuron

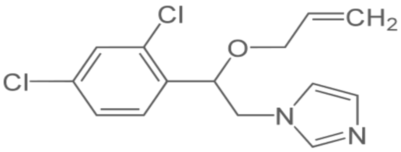

Imazalil

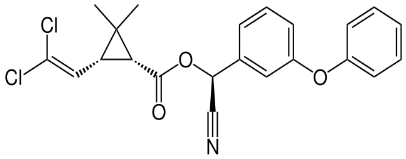

Cypermethrin

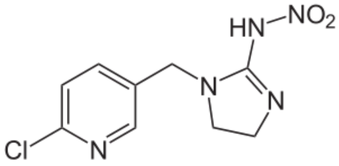

Imidacloprid

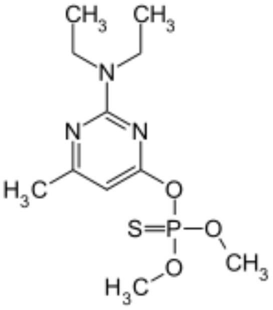

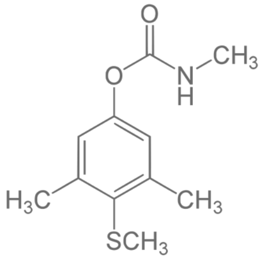

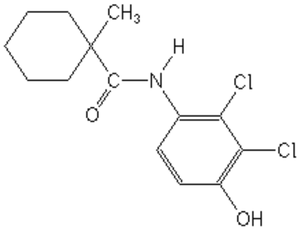

Pirimiphos-methyl | **Methiocarb** | **Fenhexamid**

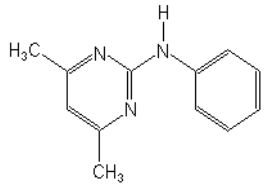

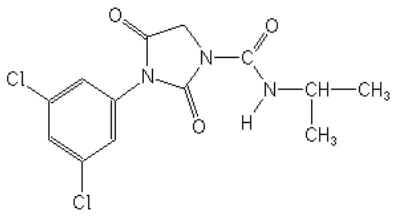

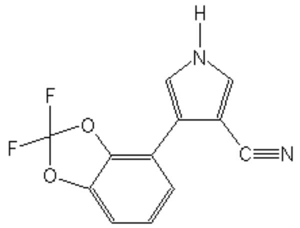

Pyrimethanil | **Iprodione** | **Fludioxonil**

<u>Pesticides tested but not inhibiting PGD2 synthesis in SC5 cells</u>

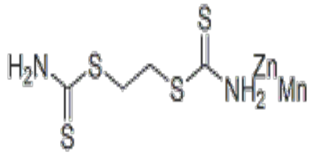

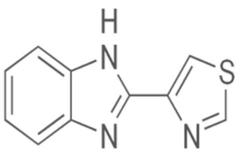

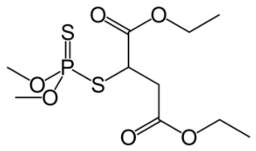

Mancozeb | **Thiabendazole** | **Malathion**

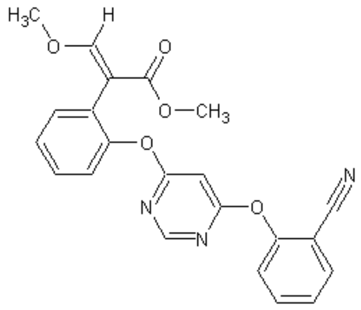

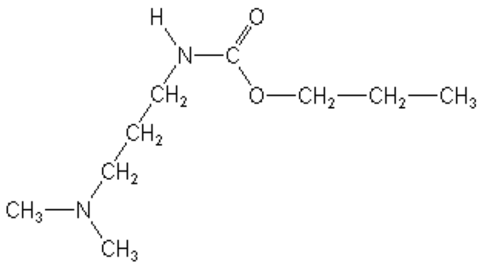

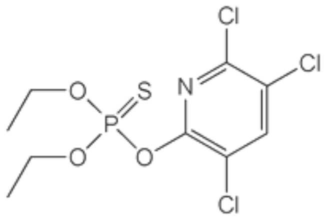

Azoxistrobin **Propamocarb** **Chlorpyrifos**

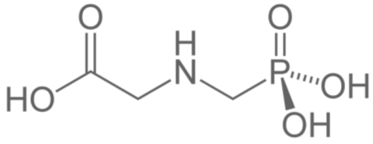

Glyphosate

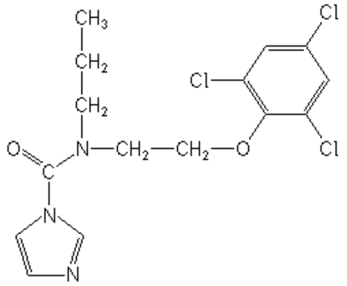

Prochloraz

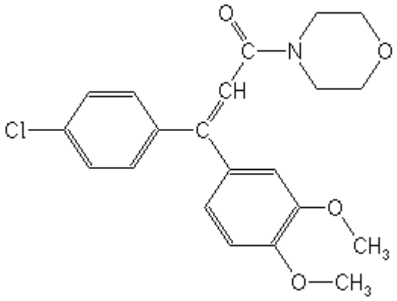

Dimethomorph

<u>Common NSAIDs</u>

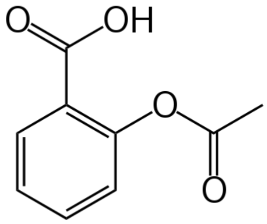

Aspirin

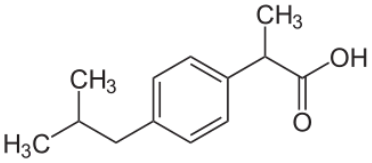

Ibuprofen

Figure S2: Suppression of PGD2 synthesis by pesticides in the mouse Sertoli cell assay

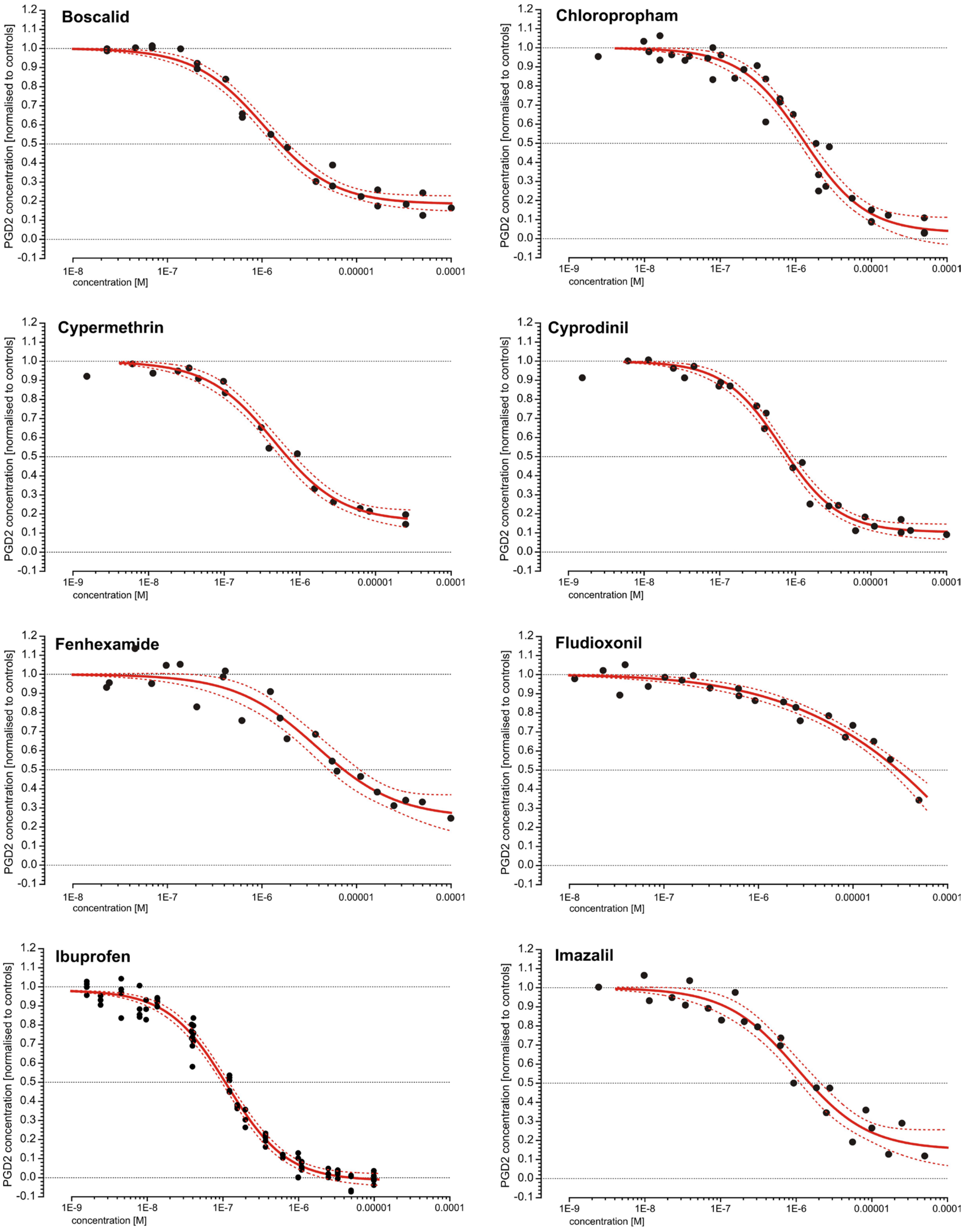

Figure S2 (contd):

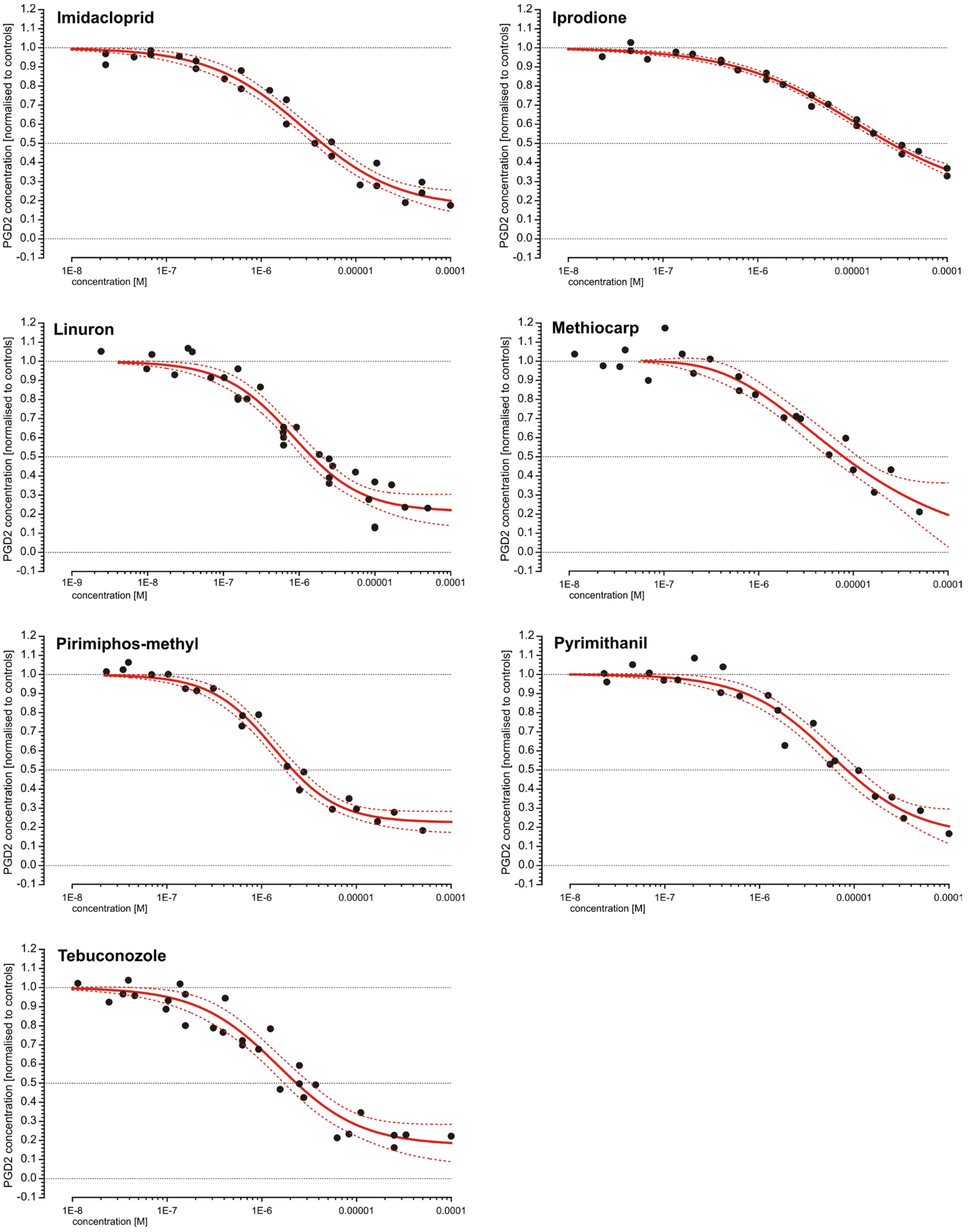

The graphs show data, best-fitting models (solid line) and 95% confidence belts (dashes line) for PGD2 synthesis inhibiting responses in SC5 (mouse Sertoli) cells after 24 hours exposure (3 replicates). All data were normalised to those of solvent controls.

Figure S3: Lack of cytotoxicity of test agents in the concentration ranges associated with PGD2 suppression

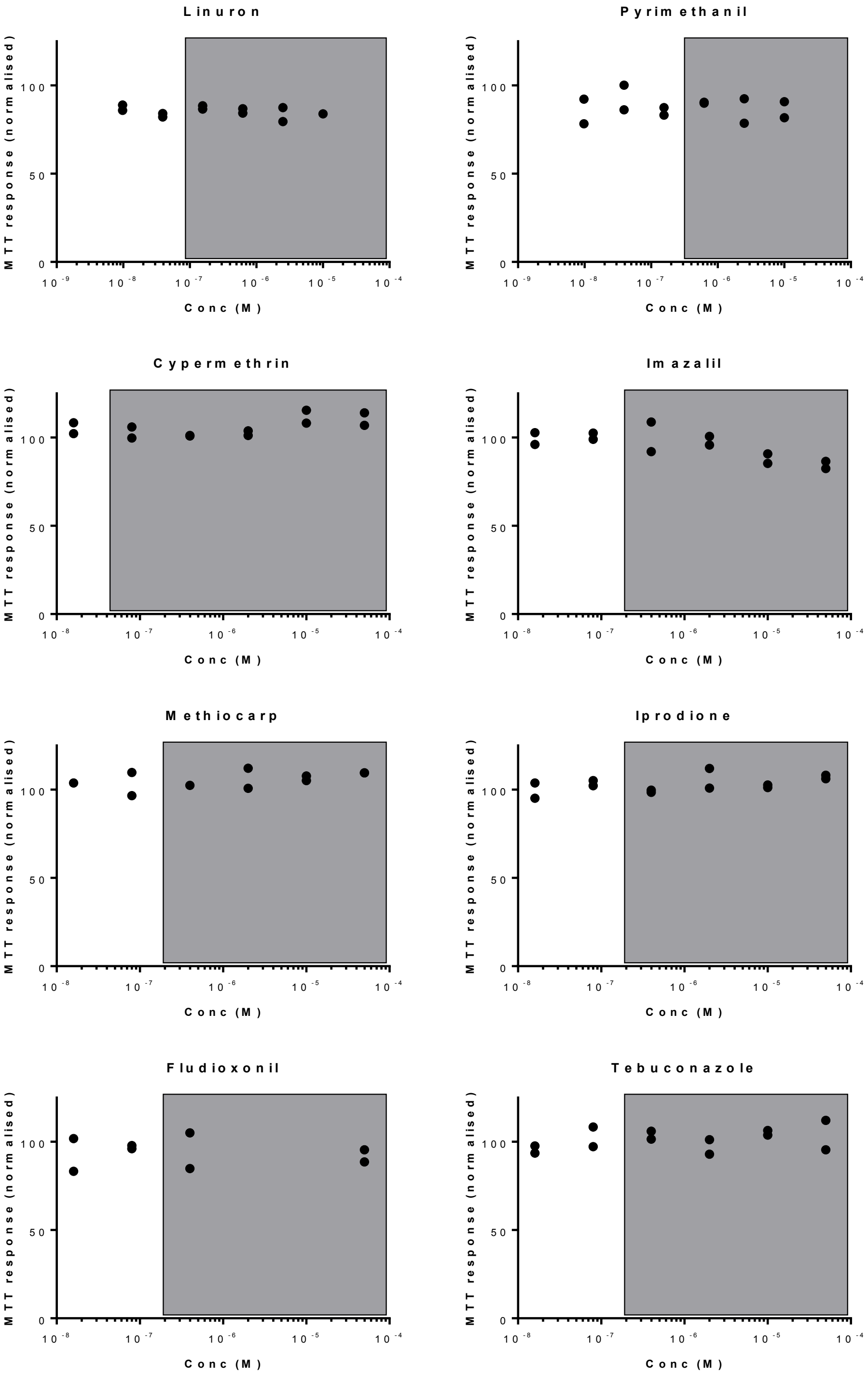

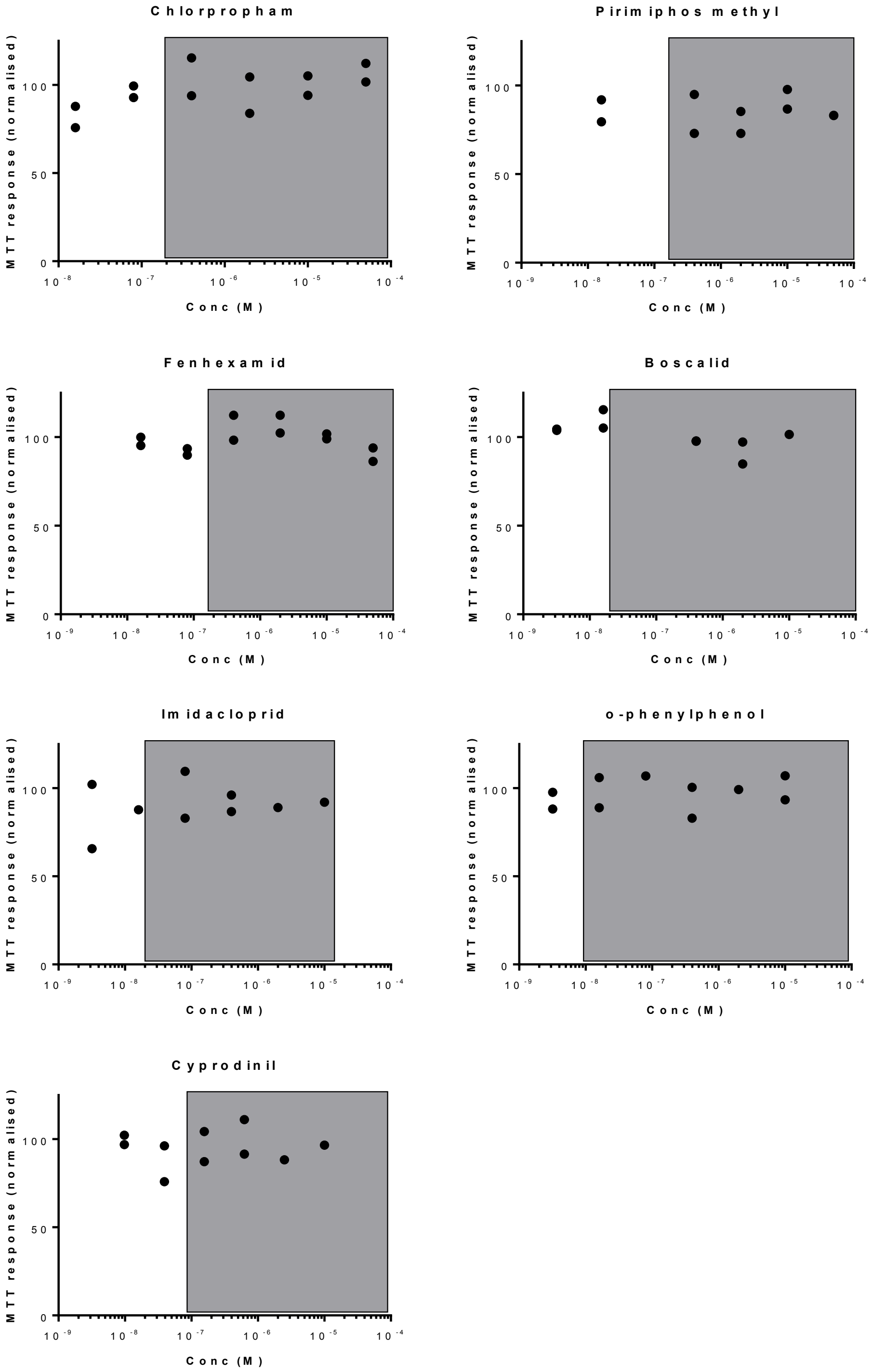

Shown are MTT responses (absorbance at 570 nm) in SC5 cells normalised to solvent-treated controls (absorbance readings in controls were set to 100%). The range associated with PGD2 suppression is depicted by the grey boxes. A clear downward trend in responses was not seen over the concentration ranges investigated.

Figure S4: Time course of PGD2 suppression in SC5 cells by OPP, ibuprofen and aspirin

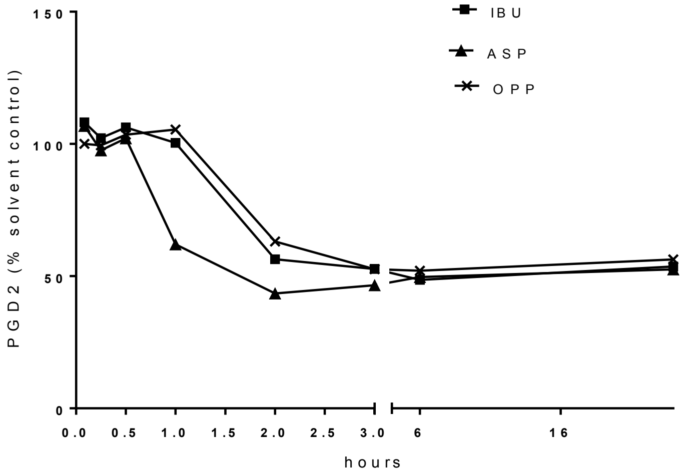

SC5 cells were exposed to ibuprofen (IBU), aspirin (ASP) and o-phenylphenol (OPP) at their respective IC50 concentrations (128 nM, 3426 nM and 175 nM of ibuprofen, aspirin and OPP, respectively) for the indicated time periods, and PGD2 concentrations measured. For each time point, PGD2 levels (average of 3 experiments performed in duplicate) are expressed as percentage of solvent controls. The last time point is 24 hours.

Figure S5: Effect of AA supplementation on the inhibition of PGD2 synthesis by ibuprofen and o-phenylphenol.

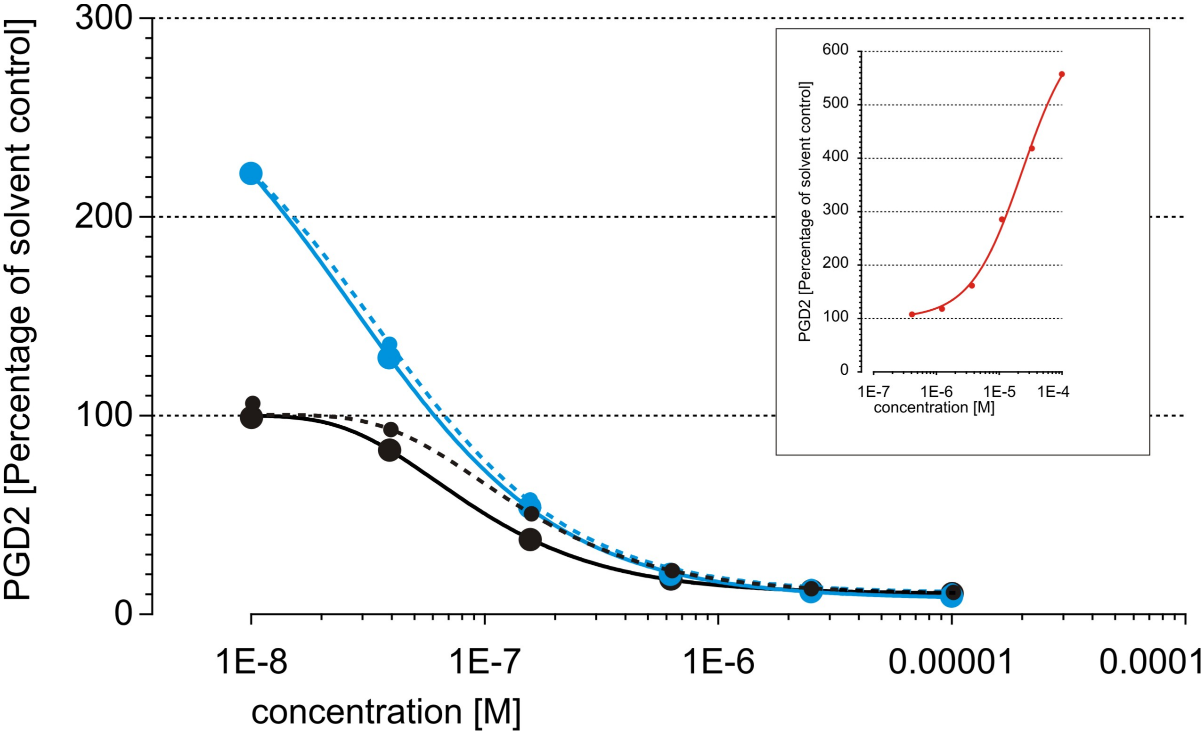

PGD2 synthesis in mouse Sertoli cells after 24 h exposure to ibuprofen (black solid line) and o-phenylphenol (black dashed line) without AA. Supplementation of AA (10 µM) during the last 2 hours of the 24 h exposure duration, with ibuprofen (blue solid line) or o-phenylphenol (blue dashed line). The inset figure (red line) shows the dose response curve of AA stimulation of PGD2 synthesis. The graphs show mean responses (dots, n=3) and their best-fitting regression models. Data were normalised against solvent controls.

Table S1: Different endocrine disrupting compounds and their IC_{50} values in PGD2 inhibition and AR-antagonism assays. Values for ethylparaben, *n*-propylparaben, *n*-butylparaben, benzophenone 3, bisphenol A, flutamide and *p,p'*-DDE (PGD2 inhibition) were obtained from Kristensen et al. (2011 b), and AR antagonism from Ermler et al. (2011). The AR antagonism data for all remaining chemicals are from Orton et al. (2011), those for PGD2 inhibition are data reported here (shown in bold).

**Compound**	**IC_{50} (M)**	
	**PGD2 inhibition**	**AR-antagonism**
Ethylparaben	7.59E-06	1.11E-04
n-Propylparaben	2.85E-06	7.01E-05
n-Butylparaben	2.43E-06	4.11E-05
Benzophenone 3	1.97E-07	1.79E-05
Bisphenol A	2.72E-06	4.07E-06
Flutamide	1.87E-06	1.56E-06
p,p'-DDE	6.11E-06	2.70E-06
**Cyprodinil**	**8.03E-07**	2.81E-05
**Imazalil**	**1.51E-06**	8.30E-06
**Pirimiphos-methyl**	**2.14E-06**	1.71E-05
**Pyrimethanil**	**8.27E-06**	9.86E-05
**Fludioxonil**	**3.02E-05**	2.62E-06
**Fenhexamid**	**7.37E-06**	7.08E-06
**Chlorpropham**	**1.34E-06**	1.92E-05
**o-Phenylphenol**	**1.75E-07**	9.57E-06
**Methiocarb**	**7.85E-06**	1.48E-05
**Tebuconazole**	**2.32E-06**	8.06E-06
**Linuron**	**1.49E-06**	6.63E-06

References

Ermler S, Scholze M, Kortenkamp A. 2011. The suitability of concentration addition for predicting the effects of multi-component mixtures of up to 17 anti-androgens with varied structural features in an in vitro AR antagonism assay. Toxicol Appl Pharmacol 257(2):189-197.

Kristensen DM, Skalkam ML, Audouze K, Lesné L, Desdoits-Lethimonier C, Frederiksen H et al. 2011b. Many putative endocrine disruptors inhibit prostaglandin synthesis. Environ Health Perspect 119(4):534-541.

Orton F, Rosivatz E, Scholze M, Kortenkamp A. 2011. Widely used pesticides with previously unknown endocrine activity revealed as in vitro antiandrogens. Environ Health Perspect 119:794-800.
